# Artificial intelligence as an independent reader of risk-dominant lung nodules: influence of CT reconstruction parameters

**DOI:** 10.1007/s00330-025-11949-8

**Published:** 2025-08-29

**Authors:** Yifei Mao, Marjolein A. Heuvelmans, Marcel van Tuinen, Donghoon Yu, Jaeyoun Yi, Matthijs Oudkerk, Zhaoxiang Ye, Geertruida H. de Bock, Monique D. Dorrius

**Affiliations:** 1 Department of Epidemiology, University Medical Center Groningen, University of Groningen, Groningen, The Netherlands; 2 Institute for Diagnostic Accuracy, Groningen, The Netherlands; 3 Department of Respiratory Medicine, Amsterdam University Medical Center, Amsterdam, The Netherlands; 4 Department of Radiology, University Medical Center Groningen, University of Groningen, Groningen, The Netherlands; 5 Coreline Soft, Seoul, Republic of Korea; 6 Department of Radiology, Tianjin Medical University Cancer Institute and Hospital, National Clinical Research Centre for Cancer, Key Laboratory of Cancer Prevention and Therapy, Tianjin’s Clinical Research Center for Cancer, Tianjin, China

**Keywords:** Solitary pulmonary nodule, X-ray computed tomography, Artificial intelligence, Computer-aided diagnosis, Classification

## Abstract

**Objectives:**

To assess the impact of reconstruction parameters on AI’s performance in detecting and classifying risk-dominant nodules in a baseline low-dose CT (LDCT) screening among a Chinese general population.

**Materials and methods:**

Baseline LDCT scans from 300 consecutive participants in the Netherlands and China Big-3 (NELCIN-B3) trial were included. AI analyzed each scan reconstructed with four settings: 1 mm/0.7 mm thickness/interval with medium-soft and hard kernels (D45f/1 mm, B80f/1 mm) and 2 mm/1 mm with soft and medium-soft kernels (B30f/2 mm, D45f/2 mm). Reading results from consensus read by two radiologists served as reference standard. At scan level, inter-reader agreement between AI and reference standard, sensitivity, and specificity in determining the presence of a risk-dominant nodule were evaluated. For reference-standard risk-dominant nodules, nodule detection rate, and agreement in nodule type classification between AI and reference standard were assessed.

**Results:**

AI-D45f/1 mm demonstrated a significantly higher sensitivity than AI-B80f/1 mm in determining the presence of a risk-dominant nodule per scan (77.5% vs. 31.5%, *p* < 0.0001). For reference-standard risk-dominant nodules (111/300, 37.0%), kernel variations (AI-D45f/1 mm vs. AI-B80f/1 mm) did not significantly affect AI’s nodule detection rate (87.4% vs. 82.0%, *p* = 0.26) but substantially influenced the agreement in nodule type classification between AI and reference standard (87.7% [50/57] vs. 17.7% [11/62], *p* < 0.0001). Change in thickness/interval (AI-D45f/1 mm vs. AI-D45f/2 mm) had no substantial influence on any of AI’s performance (*p* > 0.05).

**Conclusion:**

Variations in reconstruction kernels significantly affected AI’s performance in risk-dominant nodule type classification, but not nodule detection. Ensuring consistency with radiologist-preferred kernels significantly improved agreement in nodule type classification and may help integrate AI more smoothly into clinical workflows.

**Key Points:**

***Question*** *Patient management in lung cancer screening depends on the risk-dominant nodule, yet no prior studies have assessed the impact of reconstruction parameters on AI performance for these nodules*.

***Findings*** *The difference between reconstruction kernels (AI-D45f/1* *mm vs. AI-B80f/1* *mm, or AI-B30f/2* *mm vs. AI-D45f/2* *mm) significantly affected AI’s performance in risk-dominant nodule type classification, but not nodule detection*.

***Clinical relevance*** *The use of kernel for AI consistent with radiologist’s choice is likely to improve the overall performance of AI-based CAD systems as an independent reader and support greater clinical acceptance and integration of AI tools into routine practice*.

**Graphical Abstract:**

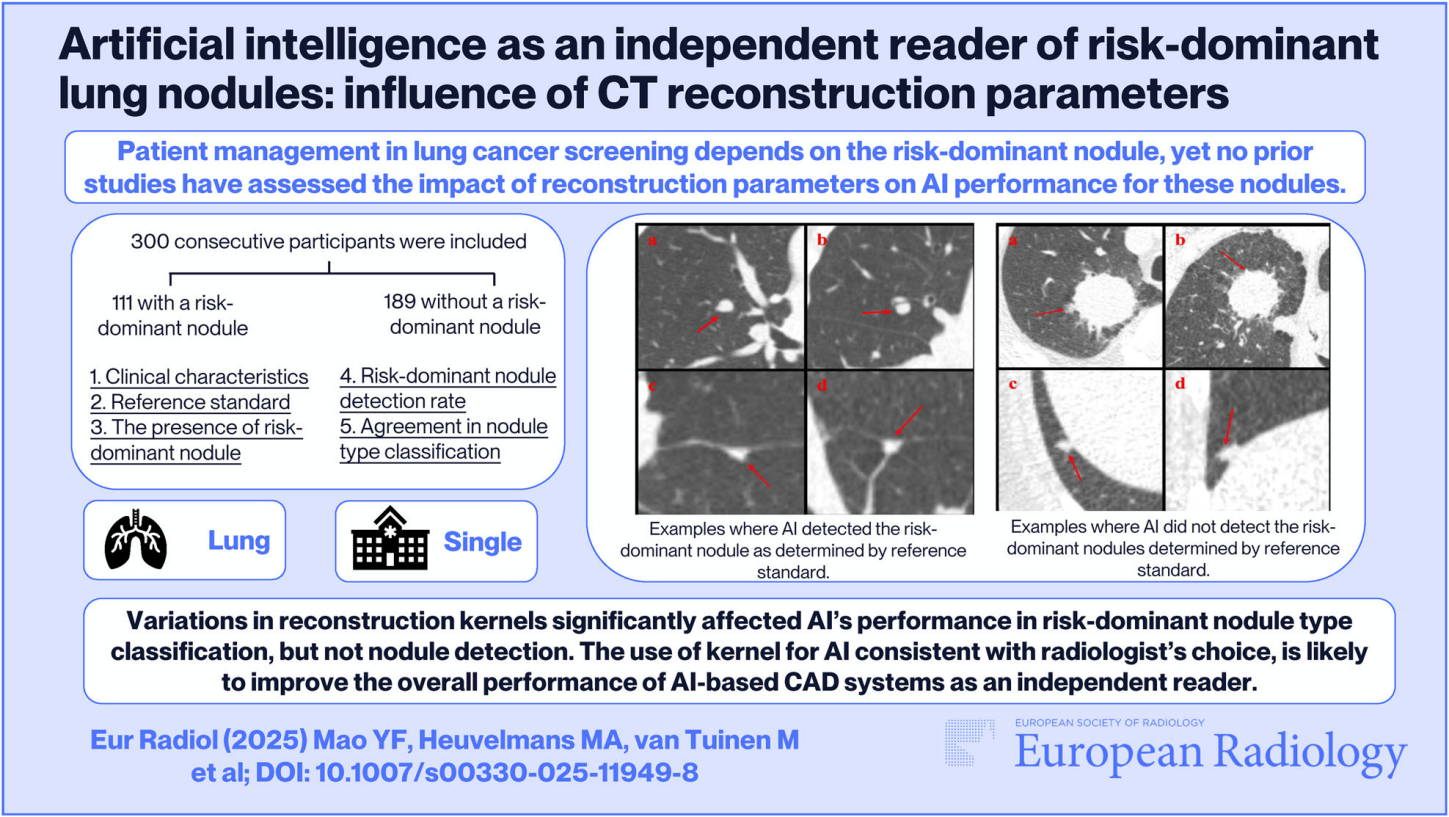

## Introduction

Lung cancer remains the leading cause of cancer-related mortality worldwide, accounting for 18% of all cancer-related deaths in 2020 [1]. Population-based lung cancer screening (LCS) programs have proven the benefits of low-dose computed tomography (LDCT) in early detection of lung cancer, significantly improving patient survival [2–4]. Consequently, an increasing number of countries are implementing LDCT LCS programs [5–8].

The role of artificial intelligence (AI) in chest CT scan workflow is becoming increasingly relevant [9–11]. AI offers the potential to improve the efficiency and accuracy of LDCT assessments. Numerous AI-based computer-aided diagnosis (CAD) systems are now available for chest CT analysis, which can automate tasks such as detection, segmentation and classification of lung nodules type (noncalcified solid, subsolid, and calcified) [9, 12]. However, certain challenges must be addressed before widespread implementation of AI as an independent reader within LDCT-based LCS programs.

Currently, most lung nodule management protocols focus on evaluating the nodule with the highest lung cancer probability based on its size, growth rate or appearance (referred to as “risk-dominant nodule” in this study) instead of analyzing all nodules to guide participant follow-up [13**–**15]. While malignancy rates of risk-dominant nodules vary across different LCS studies, for lung cancers, 80.0–97.0% were detected in these risk-dominant nodules [16, 17]. For AI-based CAD systems to function as independent readers, accurate performance of tasks must be shown for the detection of risk-dominant nodules, for nodule volume segmentation, and for the classification of risk-dominant nodule type. Both qualitative and quantitative analysis of CT scans may be significantly influenced by the choice among various reconstruction kernels, including ‘soft,’ ‘moderate,’ and ‘sharp’ [18, 19]. Previous studies have examined the influence of reconstruction parameters on various aspects of lung nodule detection [20–23] and volume categorization [24, 25], but none have centered on the risk-dominant nodule. As such, no studies have evaluated the optimal reconstruction parameters for risk-dominant nodules at the case level. Addressing these requirements is essential for integrating AI into LCS workflows.

In this study, the aim was to evaluate the effect of reconstruction parameters on the performance in risk-dominant nodule detection and type classification of an AI-based CAD software as an independent reader in a LDCT lung cancer baseline screening among a Chinese general population.

## Materials and methods

### Study cohort and CT scan protocol

Study participants were part of the Netherlands and China Big-3 (NELCIN-B3) trial, a community-based LDCT lung cancer screening program (ClinicalTrials.gov identifier: NCT03988322 and NCT03992833). The detailed study design of the NELCIN-B3 study in Tianjin has been described previously [7, 26]. Briefly, individuals were invited to undergo LDCT for lung cancer screening if they met the following inclusion criteria: (1) aged 40–74 years, (2) residents in the Hexi district of Tianjin city for at least 3 years, and (3) no self-reported history of cancer. All participants signed informed consent. Thirty-five scans were excluded due to missing reconstructions, incomplete images, or poor quality. Finally, 300 consecutive individuals who underwent baseline screening at Tianjin Medical University Cancer Institute and Hospital between June 2017 and July 2018 as part of the NELCIN-B3 cohort were included.

CT images were acquired with a 128-detector row CT scanner (Somatom Definition AS 128, Siemens Healthineers), in a low-dose setting (tube voltage: 120 kVp; reference tube current: 35 mAs). Whole chest LDCT scans were performed and reconstructed by the PACS system of CT scanners with three different combinations of section thickness, increment and kernel: 1.0 mm/0.7 mm with a medium-soft kernel of D45f (D45f/1 mm), 1.0 mm/0.7 mm with a sharp kernel of B80f (B80f/1 mm), and 2.0 mm/1.0 mm with a soft kernel of B30f (B30f/2 mm).

### Deep learning-based CAD system

A commercial deep learning-based CAD system (AVIEW LCS, v1.1.39.14, Coreline Soft) was used in this study. The deep learning algorithm used for nodule detection was trained on the public LUNA16 dataset, which includes 888 CT images with 1186 labeled nodules [27]. This algorithm employs a two-stage process: the first stage identifies nodule candidates using DenseNet and ResNet architectures, while the second stage reduces false positives using five-layer convolutional neural network models. For nodule segmentation, thresholds of −450 HU and −200 HU were applied to define the solid portion for solid and part-solid nodules, respectively. Subsolid nodules were segmented by applying a histogram-based threshold to identify ground-glass components [28]. An asymmetric deformable model was subsequently implemented to refine the segmentation of nodule regions [29]. Finally, intensity-based and texture-based radiomics features were extracted, and a random forest model that was trained with five most important features was used to classify nodules into solid, part-solid, and nonsolid types. The AI-based CAD system reports location (lobe and slice number), type (noncalcified solid, part-solid, nonsolid, and calcified), and size (diameter and volume of whole nodule, and solid component) for all identified nodules.

### AI reading

Considering the potential influence of different kernels on nodule detection, segmentation, and type classification, the reconstruction kernel should remain constant when comparing different section thickness/interval settings. In this study, the original three CT reconstruction settings (D45f/1 mm, B80f/1 mm, and B30f/2 mm) used different kernels; hence, direct comparison of images with the same kernel but different thickness/interval settings was not feasible. To address this, we performed the ‘reformation’ function in AVIEW software (Supplementary appendix 1) to process CT images initially reconstructed with a 1.0 mm/0.7 mm thickness/interval setting under the D45f kernel (D45f/1 mm), generating new CT images with a 2.0 mm/1.0 mm thickness/interval setting while maintaining the same kernel (D45f/2 mm). This reformation enabled direct investigation of the effect of thickness/interval setting through comparison between D45f/1 mm and D45f/2 mm, while keeping the reconstruction kernel constant. The reformation function in AVIEW software is based on a commonly used post-processing method, which follows the standard approach generally adopted in similar applications across PACS and imaging software. While the reformatted images may not be completely identical to those directly reconstructed from the CT scanner, such differences are minimal and unlikely to introduce significant bias. The AVIEW system then automatically detected, segmented and characterized lung nodules across the four reconstruction settings (AI-D45f/1 mm, AI-D45f/2 mm, AI-B80f/1 mm, and AI-B30f/2 mm).

### Radiologists’ reading

All 300 included CT images were independently read by two radiologists (M.D., with over 10 years of CT experience, and Y.M., with 5 years of CT experience). They manually detected lung nodules and determined the nodule type. For 3D semi-automatic volume measurements, radiologist 1 (M.D.) used Syngo.via VB30A software (MM Oncology application, Siemens Healthineers) and radiologist 2 (Y.M.) used AVIEW LCS v1.1.39.14 software to segment each detected nodule. Both readers were experienced in using the software algorithms and were blinded to AI’s and each other’s reading results and characteristics of the study population. In concordance with the NELCIN-B3 protocol, both readers used maximum intensity projection (MIP) reconstruction under the D45f kernel with 10-mm slice thickness for nodule detection. For nodule volumetric measurement, multiplanar reconstruction (MPR) under the B80f kernel with 1-mm slice thickness was applied. Any nodule with a volume ≥ 30 mm^3^ was recorded, and if multiple nodules existed, the 10 largest nodules were recorded.

### Risk-dominant nodule and reference standard

In this study, only noncalcified solid and part-solid nodules were evaluated to identify the risk-dominant nodule per CT scan. For CTs with noncalcified solid or part-solid nodules ≥ 30 mm³ (referred to collectively as solid component nodules), the nodule with the largest solid component was selected as the risk-dominant nodule (See Fig. S1). Individuals without solid component nodules meeting the ≥ 30 mm^3^ threshold were categorized as having no risk-dominant nodule. For all 300 CT scans, it was assessed whether a risk-dominant nodule was present. This step was done independently by both the radiologists and AI. Furthermore, for AI, this was assessed under four different reconstruction settings (AI-D45f/1 mm, AI-B80f/1 mm, AI-B30f/2 mm, and AI-D45f/2 mm).

All discrepancies in risk-dominant nodule selection and type classification between the two radiologists were jointly reviewed by them using Syngo.via VB30A software, to determine the presence of a risk-dominant nodule, identify which nodule was risk-dominant, and classify the nodule type. Consensus was reached through discussion, and the consensus results served as the reference standard to evaluate AI’s performance.

### Statistical analysis

All statistical analyses were performed using SPSS Statistics (IBM Corp., version 28), with a *p*-value of < 0.05 considered statistically significant. The characteristics of the study population were first described. Categorical variables were reported as numbers and percentages, while continuous variables were presented by their mean ± standard deviation.

As a first analytical step, it was determined whether scans identified as having a risk-dominant nodule by the reference standard were also identified as having a risk-dominant nodule by the four settings of the AI (AI-D45f/1 mm, AI-B80f/1 mm, AI-B30f/2 mm, and AI-D45f/2 mm), regardless of whether the same risk-dominant nodule was detected. Inter-reader agreement was calculated using Cohen’s κ statistic. Furthermore, the sensitivity and specificity of AI on the four reconstruction settings in scan-level risk-dominant nodule detectability were calculated by using reference standard and compared between different reconstruction kernels (AI-D45f/1 mm vs. AI-B80f/1 mm, and AI-B30f/2 mm vs. AI-D45f/2 mm), and between different thickness/interval settings (AI-D45f/1 mm vs. AI-D45f/2 mm), using the McNemar test.

Second, in a case-by-case assessment, it was determined whether the risk-dominant nodule identified by reference standard was also detected by the AI. We counted the absolute number and percentage of CT scans where a risk-dominant nodule determined by reference standard was also detected by AI, regardless of whether AI considered it to be the risk-dominant nodule. Comparisons of AI results between different reconstructions were performed using the McNemar test. Subsequently, for cases where AI successfully detected the reference-standard risk-dominant nodule, it was determined whether this nodule was AI’s largest nodule. This step aimed to select the matched nodules for evaluating AI’s performance in nodule type classification. Finally, for those matched nodules, the extent of agreement in nodule type classification was evaluated. This evaluation was based on reference-standard risk-dominant nodules, which consisted of only two nodule types classified by the reference standard: noncalcified solid and part-solid. However, AI’s nodule type classification included four categories (noncalcified solid, part-solid, nonsolid, calcified), as these matched nodules were not necessarily AI’s risk-dominant nodules. Thus, we could not assess the agreement in nodule type classification between AI and reference standard using a conventional n × n contingency table. Instead, the absolute number and percentage of cases where there was agreement in nodule type classification between AI and reference standard were calculated and compared between different kernels or different thickness/interval settings using the Chi-squared test.

## Results

### Description of participants

Of the 300 included participants, mean age was 61.2 years ± 7.1 (range: 40–74 years), and 159 (53.0%) were women. About 35.6% (107/300) participants were current or former smokers, and the mean number of pack-years smoked was 22.7 ± 19.0 (Table 1).

**Table 1:** Description of participants

Variable	Number (%)
Total number of participants	300 (100%)
Sex	
Women	159 (53.0%)
Men	140 (46.7%)
Unknown	1 (0.3%)
Smoking status	
Never-smokers	193 (64.3%)
Current or former	103 (34.4%)
Unknown	4 (1.3%)
Pack-years for ex- or current smokers, Mean ± SD	22.7 ± 19.0

### Reference standard and inter-radiologist agreement

Of the 300 participants, 111 (37.0%) were identified as having a risk-dominant nodule by the consensus reference standard. Radiologist 1 identified 114 risk-dominant nodules, while Radiologist 2 identified 101. Inter-radiologist agreement for the presence of a risk-dominant nodule was almost perfect (κ = 0.84, 95% CI: 0.77–0.90). The two radiologists selected the same risk-dominant nodules in 85 cases, among which no discrepancies were observed in nodule type classification. For more details of reading results by the two radiologists, see Supplementary Appendix 2.

### Presence of a risk-dominant nodule at the scan level

Concerning the presence of a risk-dominant nodule at the CT scan level, AI demonstrated varying levels of inter-reader agreement with reference standard, with Cohen’s κ values ranging from 0.25 (95% CI: 0.15–0.36) to 0.65 (95% CI: 0.56–0.74). AI-D45f/1 mm, AI-B30f/2 mm and AI-D45f/2 mm achieved a moderate to substantial agreement with the reference standard, but AI-B80f/1 mm only achieved a poor to fair agreement (Table 2). In addition, AI-D45f/1 mm showed significantly higher sensitivity (77.5% vs. 31.5%, *p* < 0.0001) and a slightly lower specificity (87.3% vs. 91.0%, *p* = 0.19) in determining the presence of a risk-dominant nodule per scan, compared to AI-B80f/1 mm. However, no significant differences were observed in sensitivities and specificities between AI-B30f/2 mm and AI-D45f/2 mm (sensitivities: 74.8% vs. 74.8%, *p* = 1.00; specificities: 85.7% vs. 85.2%, *p* = 0.76), or between AI-D45f/1 mm and AI-D45f/2 mm (sensitivities: 77.5% vs. 74.8%, *p* = 0.32; specificities: 87.3% vs. 85.2%, *p* = 0.20) (Table 2).

**Table 2:** Assessment of presence of any risk-dominant nodule at the level of a scan: AI compared with reference standard (*n* = 300)

	Assessment of reference standard				
	Scan with risk-dominant nodule (*N* = 111)	Scan without risk-dominant nodule (*N* = 189)	Scan-level agreement^*^	Sensitivity	Specificity
AI-D45f/1 mm			0.65 (0.56–0.74)	77.5%	87.3%
Scan with risk-dominant nodule	86	24			
Scan without risk-dominant nodule	25	165			
AI-B80f/1 mm			0.25 (0.15–0.36)	31.5%	91.0%
Scan with risk-dominant nodule	35	17			
Scan without risk-dominant nodule	76	172			
AI-B30f/2 mm			0.60 (0.51–0.70)	74.8%	85.7%
Scan with risk-dominant nodule	83	27			
Scan without risk-dominant nodule	28	162			
AI-D45f/2 mm			0.60 (0.51–0.69)	74.8%	85.2%
Scan with risk-dominant nodule	83	28			
Scan without risk-dominant nodule	28	161			

* Agreement for the presence of a risk-dominant nodule at the scan level between AI and reference standard was evaluated by using a Cohen’s κ statistic

### Detection of the risk-dominant nodules

**Fig. 1:**
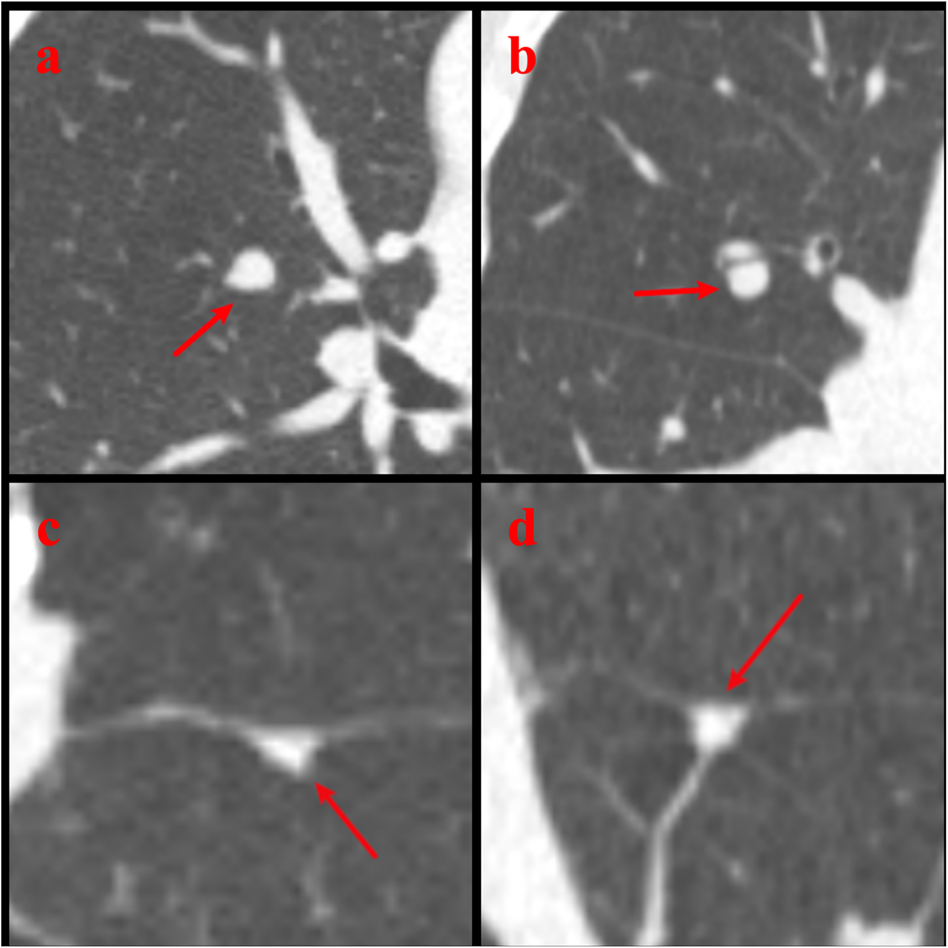
Examples where AI detected the risk-dominant nodule as determined by reference standard. Example 1. LDCT LCS images from a 70-year-old female. **a** Axial (D45f/1 mm, lung window) and (**b**) coronal (D45f/1 mm, lung window) LDCT images show a vessel-attached lesion in the right upper lobe (lesion 2). The reference standard determined this lesion as the risk-dominant nodule and classified it as noncalcified solid. AI on all four reconstruction settings detected this nodule and agreed on the nodule type classification with the reference standard. This nodule was the largest nodule for AI-D45f/1 mm, AI-B80f/1 mm, and AI-D45f/2 mm, but not for AI-B30f/2 mm. Example 2. LDCT LCS images from a 69-year-old female. **c** Axial (D45f/1 mm, lung window) and (**d**) coronal (D45f/1 mm, lung window) LDCT images show a fissure-attached lesion in the left lower lobe (lesion 1). The reference standard determined this lesion as the risk-dominant nodule and classified it as noncalcified solid. AI on all four reconstruction settings detected this nodule, and this nodule was the largest nodule for AI on all four reconstruction settings. AI-D45f/1 mm and AI-D45f/2 mm agreed on the nodule type classification with the reference standard, while AI-B80f/1 mm misclassified it as calcified, and AI-B30f/2 mm misclassified it as nonsolid. LCS, lung cancer screening; LDCT, low-dose CT

**Fig. 2:**
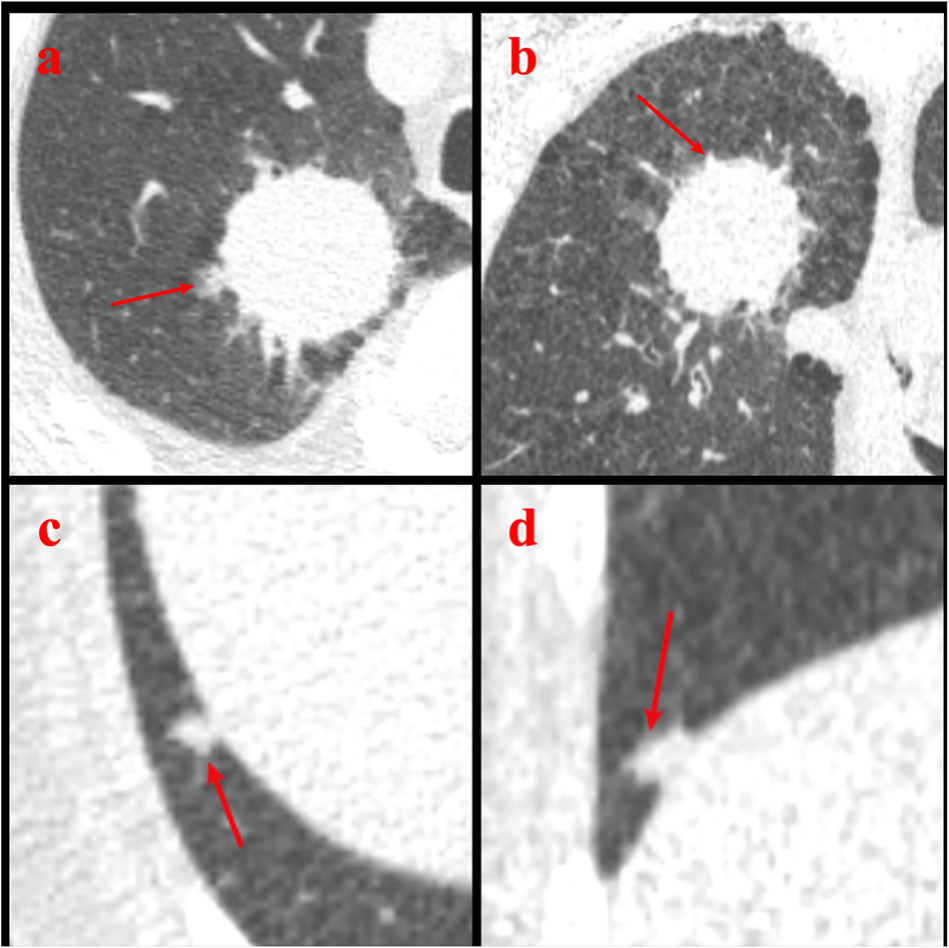
Examples where AI did not detect the risk-dominant nodules determined by reference standard. Example 1. LDCT LCS images from a 65-year-old male. **a** Axial (D45f/1 mm, lung window) and (**b**) coronal (D45f/1 mm, lung window) LDCT images show a vessel-attached lesion in the right upper lobe. The reference standard determined this lesion as the risk-dominant nodule and classified it as noncalcified solid (total volume = 39,810.0 mm^3^). However, AI on all four reconstruction settings did not mark this lesion as a nodule, because the lesion larger than 30,000 mm^3^ in volume was regarded as a mass and not segmented by the AI algorithm. Example 2. LDCT LCS images from a 67-year-old male. **c** Axial (D45f/1 mm, lung window) and (**d**) coronal (D45f/1 mm, lung window) LDCT images show a pleura-attached lesion in the right lower lobe. The reference standard determined this lesion as the risk-dominant nodule and classified it as noncalcified solid (total volume = 90.0 mm^3^). However, AI on all four reconstruction settings did not mark this lesion as a nodule. LCS, lung cancer screening; LDCT, low-dose CT

In the case-by-case analysis, it turned out that AI detected the risk-dominant nodules identified by the reference standard in 82.0% (91/111) to 87.4% (97/111) of cases (Table 3 and Figs. 1, 2). No significant differences were observed in AI’s detection rate of nodules, that were specified as risk-dominant nodules by the reference standard, between different kernels (D45f/1 mm vs. B80f/1 mm, *p* = 0.26; AI-B30f/2 mm vs. D45f/2 mm, *p* = 0.85), or between different thickness/interval settings (D45f/1 mm vs. D45f/2 mm, *p* = 0.44). In addition, AI’s largest nodule matched the reference-standard risk-dominant nodule in 58.8% (57/97), 68.1% (62/91), 56.4% (53/94), and 59.1% (55/93) of cases for AI-D45f/1 mm, AI-B80f/1 mm, AI-B30f/2 mm, and AI-D45f/2 mm, respectively.

**Table 3:** AI’s performance in detecting and characterizing the risk-dominant nodules identified by reference standard (*n* = 111)

	AI-D45f/1 mm vs. reference standard	AI-B80f/1 mm vs. reference standard	AI-B30f/2 mm vs. reference standard	AI-D45f/2 mm vs. reference standard
AI detected the risk-dominant nodule identified by reference standard, *n*/111, % (95% CI)	97/111, 87.4% (79.7–92.9%)	91/111, 82.0% (73.6–88.6%)	94/111, 84.7% (76.6–90.8%)	93/111, 83.8% (75.6–90.1%)
Reference-standard risk-dominant nodule is the largest nodule for AI	57/97, 58.8%	62/91, 68.1%	53/94, 56.4%	55/93, 59.1%
Same nodule type	50	11	32	43
Different nodule type	7	51	21	12
Reference-standard risk-dominant nodule is not the largest nodule for AI	40/97, 41.2%	29/91, 31.9%	41/94, 43.6%	38/93, 40.9%

### Agreement in nodule type classification

Of these matched nodules, AI-D45f/1 mm exhibited a significantly higher proportion of agreement with the reference standard in nodule type classification compared to AI-B80f/1 mm (87.7% [50/57] vs. 17.7% [11/62], *p* < 0.0001). AI-B30f/2 mm showed a lower proportion of agreement in nodule type classification compared to AI-D45f/2 mm (60.4% [32/53] vs. 78.2% [43/55], *p* = 0.04). In addition, no significant difference was observed in the proportion of agreement with the reference standard in nodule type classifications between AI-D45f/1 mm and AI-D45f/2 mm (87.7% [50/57] vs. 78.2% [43/55], *p* = 0.18) (Table 4 and Fig. 1a, b). Regarding disagreements, AI-B80f/1 mm more frequently misclassified noncalcified solid nodules as calcified (46/62, 74.2%), and AI-B30f/2 mm more often misclassified noncalcified solid nodules as nonsolid (16/53, 30.2%) (Table 4 and Fig. 1c, d).

**Table 4:** Agreement and disagreement in nodule type classifications between AI and reference standard

AI detected the risk-dominant nodule identified by reference standard	AI-D45f/1 mm (*N* = 97)	AI-B80f/1 mm (*N* = 91)	AI-B30f/2 mm (*N* = 94)	AI-D45f/2 mm (*N* = 93)
Reference-standard risk-dominant nodule is the largest nodule for AI	57/97	62/91	53/94	55/93
Agreement in nodule type	50	11	32	43
Correct classification of noncalcified solid	49	11	30	41
Correct classification of part-solid	1	0	2	2
Disagreement in nodule type	7	51	21	12
Misclassification of noncalcified solid	3	46	18	9
Misclassified as part-solid	1	0	2	1
Misclassified as nonsolid	2	0	16	8
Misclassified as calcified	0	46	0	0
Misclassification of part-solid	4	5	3	3
Misclassified as noncalcified solid	3	2	3	2
Misclassified as nonsolid	1	0	0	1
Misclassified as calcified	0	3	0	0

## Discussion

In this study, we evaluated the influence of different reconstruction parameters on the performance of AI as an independent reader in a baseline LDCT screening among a Chinese general population. Our findings indicate that differences in reconstruction kernels could impact AI’s sensitivity but not specificity in determining the presence of a risk-dominant nodule at the scan level, especially when comparing medium-soft to sharp kernels. For reference-standard risk-dominant nodules, AI’s nodule detection rate was not significantly affected by variations in reconstruction kernels. In contrast, different choices of reconstruction kernels had a significant influence on the proportion of agreement in nodule type classification between AI and reference standard. Additionally, a change in the section thickness/interval did not substantially impact AI’s performance in any of the three aspects: determining the presence of a risk-dominant nodule at the scan level, nodule detection, or nodule type classification.

According to the current lung nodule management guidelines, scans without a risk-dominant nodule are generally considered as negative screens, and the patients are recommended for the next round of screening without a short-term follow-up [13, 14]. Our findings indicated that the change in reconstruction kernels could significantly affect AI’s sensitivity in determining the presence of a risk-dominant nodule at the case level, and thus the patient management. In contrast, the change in slice thickness/interval from 1.0 mm/0.7 mm to 2.0 mm/1.0 mm did not substantially influence AI’s sensitivity and thus the patient management. Notably, AI applied to a hard kernel (AI-B80f/1 mm) had the lowest sensitivity and lowest scan-level agreement with reference standard, compared to AI on other reconstruction settings. In majority (58/76) of false-negative results by AI-B80f/1 mm, although AI-B80f/1 mm detected reference-standard risk-dominant nodule, it misclassified these nodules as calcified, thus falsely rating the scan as having no risk-dominant nodule.

For human readers in clinical practice, soft or medium-soft kernels are commonly used for lung nodule detection, as they are optimized for visualizing soft tissues. However, prior studies reported no significant difference in nodule detectability for the CAD systems between reconstruction kernels, and their comparisons were on a per-nodule basis [21, 23]. Hwang et al compared the performance of a CAD system across three different reconstruction kernels (standard [B], sharp [C], lung enhanced [L]) in 36 lung screening participants. They demonstrated little variation in nodule detection rate between the kernels, and no significant difference was observed [21]. Ebner et al investigated the impact of different reconstruction kernels (i30, i50, and i70) on the performance of two CAD systems using 133 solid and 133 nonsolid nodules in 55 phantoms. Their findings showed no significant difference in nodule detection sensitivity between three reconstruction kernels (*p* > 0.8) for either CAD system [23]. In agreement with prior studies, our findings, focused on risk-dominant nodules, revealed that differences in reconstruction kernels did not significantly affect the nodule detection rate on a per scan basis.

Regarding the influence of section thickness/interval settings on lung nodule detectability, Kim et al performed a CAD program on CT images with three reconstruction settings (thickness/interval: 1 mm/1 mm, 5 mm/1 mm, and 5 mm/1 mm) from 10 patients with lung nodules. Their findings showed that the sensitivity of nodule detection improved with thinner reconstruction thickness (95.2% vs. 94.2%) and smaller intervals (94.2% vs. 88.6%), although without *p*-values provided [20]. Similarly, our results demonstrated that AI on the thinner images (AI-D45f/1 mm) achieved a slightly higher risk-dominant nodule detection rate compared to thicker images (AI-D45f/2 mm) (87.4% vs. 83.8%). Improvement in risk-dominant nodule detectability was in line with the theory that thinner images reduce partial volume artifact, improve image resolution, and increase the visibility of small features, thereby increasing lung nodule detectability [30]. However, in our study, the decrease in thickness/interval from 2.0 mm/1.0 mm to 1.0 mm/0.7 mm only showed a small non-significant improvement in risk-dominant nodule detection rate.

To the best of our knowledge, no previous studies have examined the impact of reconstruction kernel or slice thickness/interval settings on pulmonary nodule type classification by a CAD system. Our findings revealed that differences in reconstruction kernels, particularly between medium-soft and sharp kernels, significantly influenced the proportion of agreement in lung nodule type classification between AI and reference standard, while different slice thickness/interval settings did not have a significant impact. In this study, both radiologists used the kernel of D45f to determine lung nodule type. The AI-based CAD system using the same reconstruction kernel as radiologists outperformed AI using other different reconstruction kernels in nodule type classification (AI-D45f/1 mm vs. AI-B80f/1 mm, and AI-D45f/2 mm vs. AI-B30f/2 mm). This suggests that using the same reconstruction kernel for both AI and radiologists significantly increases agreement in nodule type classification, likely due to shared visual characteristics in image texture and structure. While this consistency is not strictly required from a technical perspective, it can enhance radiologists’ confidence in AI’s output and facilitate smoother integration of AI into clinical workflow.

Notably, slice thicknesses > 2 mm were not assessed in this study. Current protocols, such as the 2017 Fleischner Society guidelines [15], recommend thin-slice CT images (≤ 1.5 mm) for accurate detection and characterization of small pulmonary nodules. Accordingly, our study compared only 1 mm and 2 mm thin-section reconstructions, which are commonly used in clinical or LCS settings. The potential impact of thicker reconstructions (e.g., 3 mm or greater) on nodule detection and nodule type classification remains uncertain and warrants further investigation.

This study has several limitations. First, in this study, we had no access to the data on the pathological diagnosis of lung cancer. Instead, we applied the consensus read by two radiologists as a reference standard. Future studies incorporating histopathological data are warranted to externally validate our findings and improve their clinical applicability. Second, the AI algorithm was initially developed specifically to detect pulmonary nodules, generally defined as a lesion < 3 cm in diameter (approximately 14,137 mm^3^ in volume). Therefore, a conservative upper volume threshold of 30,000 mm³ was applied to minimize false positives by AI, which may result in missed detection of rare but clinically relevant lesions with > 3 cm diameter, such as early-stage invasive lung cancer. Future versions of the AI algorithm may need to be optimized to detect larger lesions as well. Lastly, the lung nodule detection and type classification were performed using a single AI algorithm in an automatic way. Further studies are needed to investigate whether different AI algorithms would support our findings on different reconstruction parameters.

In conclusion, variations in reconstruction kernel, especially differences between medium-soft and sharp kernels, significantly affected the risk-dominant nodule type classification rate, but not the detection rate. AI applied to a hard kernel should not be considered due to the poor performance in nodule type classification. Increasing slice thickness/interval from 1.0/0.7 mm to 2.0 mm/1.0 mm made no substantial difference in either automated risk-dominant nodule detection or type classification at the case level by the CAD software. Based on our findings and clinical preference, using a consistent kernel as the radiologist’s choice is likely to improve the overall performance of CAD systems as an independent reader and support greater clinical acceptance and integration of AI tools into routine practice.

## Supplementary information

ELECTRONIC SUPPLEMENTARY MATERIAL

## Abbreviations

CAD: Computer-aided diagnosis
LCS: Lung cancer screening
LDCT: Low-dose CT
NELCIN-B3: Netherlands and China Big-3
