# Supplementary material for: Artificial intelligence as an independent reader of risk-dominant lung nodules: influence of CT reconstruction parameters
Source: Eur Radiol. 2025 Aug 29;36(3):2014–23. doi: 10.1007/s00330-025-11949-8 (PMC12963105; doi:10.1007/s00330-025-11949-8)
Supplement: Supplementary file 1 — ELECTRONIC SUPPLEMENTARY MATERIAL [file 330_2025_11949_MOESM1_ESM.pdf]

Artificial Intelligence as an independent Reader of Risk-dominant Lung Nodules: Influence of CT Reconstruction Parameters

ELECTRONIC SUPPLEMENTARY MATERIAL

Appendix 1. Reformatting algorithm for reconstruction slice thickness and interval

We use a Ray-sum projection-based reformatting algorithm. This method works based on the principles of ray projection through the volumetric data to resample and aggregate the information across different slices. To reformat the slice thickness from 1mm to 2mm, we applied a sum-based Ray-sum projection algorithm. This method projects pixel intensities along the z-axis, summing the intensities across adjacent slices to create new thicker slices. For the slice interval reformatting from 0.7mm to 1mm, we applied interpolation techniques to resample the data. A trilinear interpolation method was used to generate additional slices at the new 1mm intervals.

Appendix 2. Details about reading results of two radiologists.

Radiologist 1 detected 197 solid-component nodules with ≥ 30 mm^3 volume, among which, 114 were identified as risk-dominant nodules (median volume: 73 mm^3 [IQR: 46-144 mm^3]), and the remaining 83 were not considered as risk-dominant (median volume: 47 mm^3 [IQR: 36-75 mm^3]). Radiologist 2 detected 150 solid-component nodules with ≥ 30 mm^3 volume, among which, 101 were identified as risk-dominant nodules (median volume: 56 mm^3 [IQR: 36-112 mm^3]), and the remaining 49 were not considered as risk-dominant (median volume: 42 mm^3 [IQR: 36-71 mm^3]).

Figure S1. Workflow for selection of risk-dominant nodules.

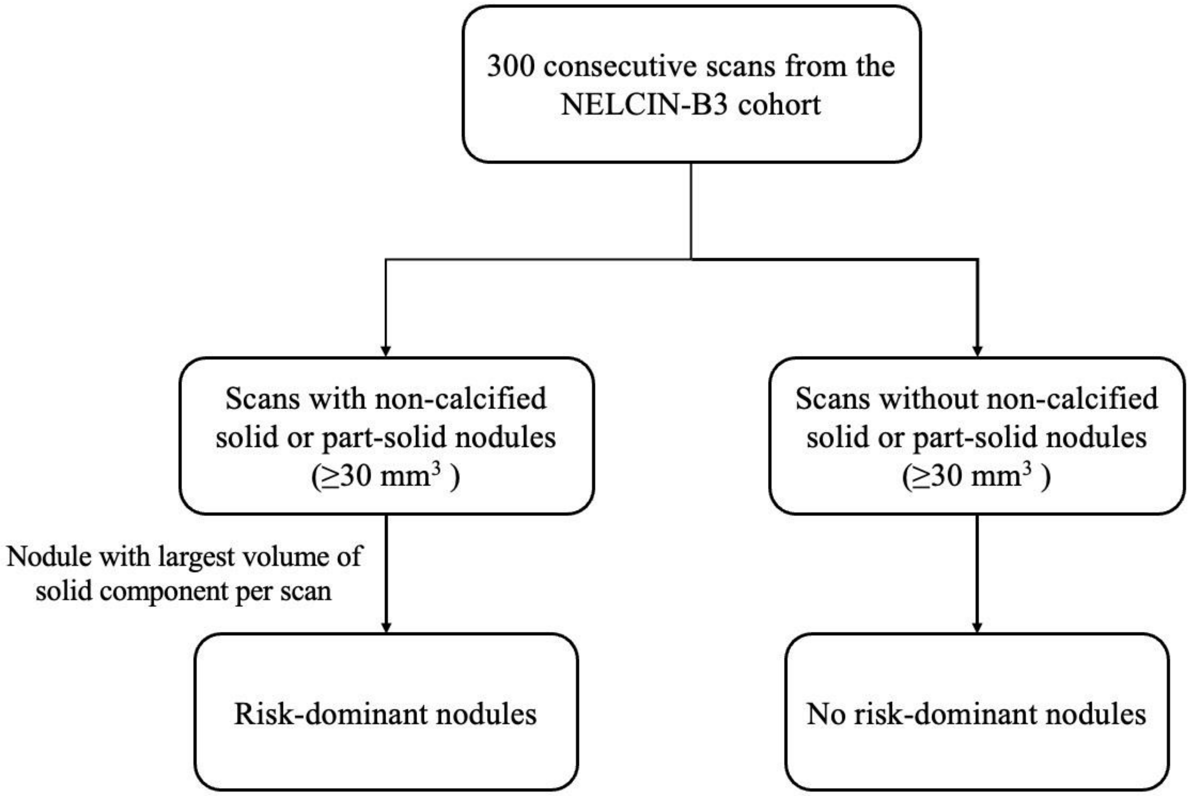

Note: Risk-dominant nodule was defined as the non-calcified solid or part-solid nodule with the largest solid component (≥30 mm^3) in each CT.
